# Lipidomic profiling of plasma free fatty acids in type-1 diabetes highlights specific changes in lipid metabolism

**DOI:** 10.1016/j.bbalip.2020.158823

**Published:** 2021-01

**Authors:** Amélie I.S. Sobczak, Samantha J. Pitt, Terry K. Smith, Ramzi A. Ajjan, Alan J. Stewart

**Affiliations:** a School of Medicine, University of St Andrews, Medical and Biological Sciences Building, St Andrews, Fife KY16 9TF, United Kingdom; b Schools of Biology and Chemistry, University of St Andrews, Biomedical Sciences Research Complex, St Andrews, Fife KY16 9ST, United Kingdom; c Leeds Institute of Cardiovascular and Metabolic Medicine, University of Leeds, Leeds LS2 9DA, United Kingdom

**Keywords:** D5D, delta-(5)-desaturase, FAME, fatty acid methyl ester, FA, fatty acid, FFA, free fatty acid, HbA1c, glycated haemoglobin A1c, GC–MS, gas chromatography–mass spectrometry, HDL, high-density lipoprotein, HSA, human serum albumin, LDL, low-density lipoprotein, SCD1, stearoyl-CoA desaturase 1, T1DM, type-1 diabetes mellitus, Fatty acid metabolism, GC–MS, HbA1c, Lipid metabolism, Lipidomics, Non-esterified fatty acid

## Abstract

Type-1 diabetes mellitus (T1DM) is associated with metabolic changes leading to alterations in glucose and lipid handling. While T1DM-associated effects on many major plasma lipids have been characterised, such effects on plasma free fatty acids (FFA) have not been fully examined. Using gas chromatography–mass spectrometry, we measured the plasma concentrations of FFA species in individuals with T1DM (*n* = 44) and age/sex-matched healthy controls (n = 44). Relationships between FFA species and various parameters were evaluated. Plasma concentrations of myristate (14:0), palmitoleate (16:1), palmitate (16:0), linoleate (18:2), oleate (18:1c9), *cis*-vaccenate (18:1c11), eicosapentaenoate (20:5), arachidonate (20:4) and docosahexanoate (22:6) were reduced in the T1DM group (*p* < 0.0001 for all, except *p* = 0.0020 for eicosapentaenoate and *p* = 0.0068 for arachidonate); α-linolenate (18:3) and dihomo-γ-linolenate (20:3) concentrations were unchanged. The saturated/unsaturated FFA ratio, n-3/n-6 ratio, *de novo* lipogenesis index (palmitate (main lipogenesis product)/linoleate (only found in diet)) and elongase index (oleate/palmitoleate) were increased in the T1DM group (*p* = 0.0166, *p* = 0.0089, *p* < 0.0001 and *p* = 0.0008 respectively). The stearoyl-CoA desaturase 1 (SCD1) index 1 (palmitoleate/palmitate) and index 2 (oleate/stearate) were reduced in T1DM (p < 0.0001 for both). The delta-(5)-desaturase (D5D) index (arachidonate/dihomo-γ-linolenate) was unchanged. Age and sex had no effect on plasma FFA concentrations in T1DM, while SCD1 index 1 was positively correlated (*p* = 0.098) and elongase index negatively correlated with age (*p* = 0.0363). HbA1c was negatively correlated with all plasma FFA concentrations measured except α-linolenate and dihomo-γ-linolenate. Correlations were observed between plasma FFA concentrations and cholesterol and HDL concentrations, but not LDL concentration or diabetes duration. Collectively, these results aid our understanding of T1DM and its effects on lipid metabolism.

## Highlights

• Plasma concentrations of major FFA species are lower in T1DM compared to controls.
• Plasma FFA concentrations negatively correlates with HbA1c in T1DM.
• The SCD1 index is reduced in T1DM.
• Lipogenesis, elongase, n3/n6, saturated/unsaturated indices are increased in T1DM.
• Collectively, the data highlight specific changes in lipid metabolism in T1DM

## Graphical abstract

**Figure:**
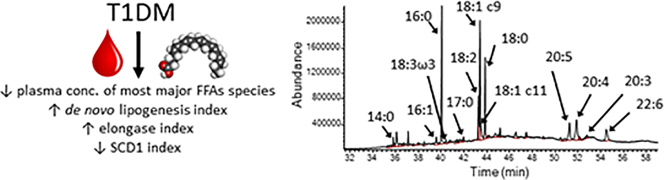

## 1 Introduction

Type-1 diabetes mellitus (T1DM) is an immune disease in which the β-cells in the islets of the pancreas are destroyed, resulting in impaired insulin secretion and control of blood glucose [1]. Worldwide incidence varies, with age-adjusted incidences ranging from 0.1/100,000 new occurrences per year in China and Venezuela to 36.5 and 36.8/100,000 new occurrences per year in Finland and Sardinia, respectively [2]. Dysregulation of plasma insulin and glucose concentrations in T1DM can lead to a range of metabolic changes [3,4]. In particular, plasma lipid composition is known to be altered in individuals with T1DM, as shown by several lipidomic studies [[5], [6], [7], [8]]. However, many of those studies focus on infants and young children, to investigate the development of T1DM, rather than the characteristics of the established disease in adults [3,[9], [10], [11], [12], [13], [14], [15]]. Moreover, the previous work has primarily focussed on lipoproteins, cholesterol and esterified fatty acids, while non-esterified or “free” fatty acids (FFAs) have had much less attention. This is because they are more sensitive to daily metabolic variations than other lipids.

FFAs are important signalling molecules and as well as being an important source of energy, impact on numerous physiological processes, including the regulation of inflammation, the management of oxidative stress, the composition of cellular membranes and signal transduction, as recently reviewed by us [16]. Thus, changes in the composition of plasma FFAs can potentially cause further complications in individuals with T1DM and, while they reflect a wider dysregulation of lipid metabolism, they can also be altered in different ways to other lipids and thus are worthy of further study in their own right [16]. Important aspects of the plasma FFA profile are the saturated/unsaturated FFA ratio and the n-3/n-6 ratio as those directly impact the physiological processes mentioned above. In addition, the concentrations of specific FFAs species can be used to calculate indices reflecting desaturation, elongation and *de novo* lipogenesis activity. Thus, to gain insight into how T1DM influences the plasma FFA profile, we measured and compared plasma concentrations of major FFA species in individuals with T1DM and an age/sex-matched control group without diabetes. In addition, we explored how plasma FFA concentrations and lipid indices compare with sex, age and the concentrations of glycated haemoglobin A1c (HbA1c) and diverse lipids.

## 2 Materials and methods

### 2.1 Clinical sample collection

A total of 44 individuals with T1DM and 44 controls were recruited from Leeds Teaching Hospital Trust following approval by the Leeds West Research Ethics Committee (REC: 09/H1307/12). We excluded individuals with a history of acute coronary syndrome or those that had experienced a stroke within 3 months of enrolment, individuals who had received prior treatment with aspirin, clopidogrel, warfarin or non-steroidal anti-inflammatory drugs or who had a current treatment with any drug other than insulin, individuals with a history of deep venous thrombosis or pulmonary embolism, previous or current history of upper gastrointestinal pathology, malignancy or coagulation disorders, individuals with abnormal liver function tests (alanine transaminase >3 fold upper limit of normal) or abnormal thyroid function tests and individuals with proteinuria, advanced nephropathy or clinical signs of neuropathy or retinopathy (except for those with background changes). Written informed consent was obtained. All blood samples were collected after a light breakfast (to avoid hypoglycaemia in T1DM individuals) and within 2 h the blood was centrifuged at 2400 ×*g* for 20 min at 4 °C. The plasma was snap frozen in liquid nitrogen and stored at −40 °C until analysis. Plasma concentration of human serum albumin (HSA), HbA1c, fasting glucose, triglycerides, cholesterol, high-density lipoprotein (HDL) and low-density lipoprotein (LDL) were measured with routine methods.

### 2.2 Measurement of plasma FAMEs

In order to characterise the FFAs present in each sample, they were converted to fatty acid methyl esters (FAMEs) and analysed by gas chromatography–mass spectrometry (GC–MS) analysis. This was performed by thawing the citrated plasma samples and spiking them with an internal standard (heptadecanoate; 17:0, 100 pmoles) to allow for normalisation. Dole's protocol was used to extract the FFAs [17], and the FFAs were converted to FAMEs by incubating them for 5 h at 45 °C with 1500 μl of methanol, 200 μl of toluene and 300 μl of 8% HCl. Nitrogen was used to evaporate the samples to dryness. The FAMEs were dissolved in 500 μl of water and 500 μl of hexane and the hexane phase was collected. The samples were left to evaporate to dryness in a fume hood. The FAMEs were then dissolved in 30 μl dichloromethane and analysed by GC–MS using 1–2 μl of the resultant samples. The instrument used was a GC-6890 N, MS detector-5973 (Agilent Technologies, Santa Clara, CA, USA) with a ZB-5 column (30 m × 25 mm × 25 mm; Phenomenex, Torrance, CA, USA). The temperature program was: at 70 °C for 10 min, followed by a gradient to 220 °C at 5 °C /min, and held at 220 °C for a further 15 min. The mass spectra were acquired from 50 to 500 amu. The FAMEs were identified by comparison of the retention time and fragmentation pattern of the samples with that of various FAME standard mixtures (Supelco, Bellefonte, PA, USA) as previously described [18].

### 2.3 Data analysis and representation

Differences between groups were analysed using multiple Student's *t*-tests or, for continuous parameters, using Pearson's correlation test. The significance threshold was set at *p* ≤ 0.05. Statistical analyses were performed and graphs were generated with Prism 8.2.1 (GraphPad Software, La Jolla, CA, USA). Data are represented as mean ± standard deviation (SD).

## 3 Results

### 3.1 Demographic information and measure of plasma concentrations of different molecules

**Fig. 1:**
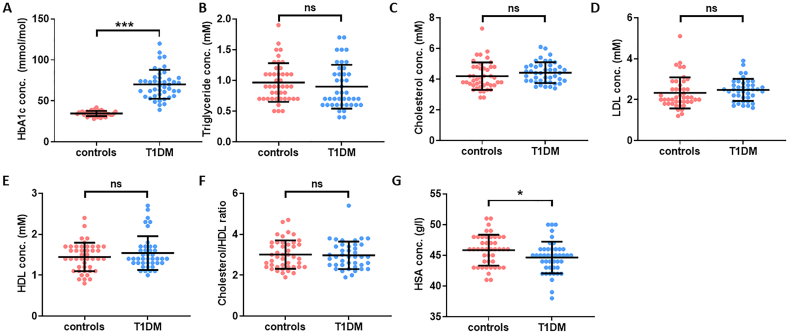
Plasma concentrations of HbA1c, lipids and HSA in T1DM and control individuals. Plasma concentrations of A. HbA1c, B. triglycerides, C. cholesterol, D. LDL, E. HDL, F. cholesterol/HDL ratio and G. HSA are presented for T1DM and controls. The concentrations of triglycerides, cholesterol, LDL and HDL and the cholesterol/HDL ratio were not different in both groups. HbA1c concentration was higher in the T1DM group (*p* < 0.0001) and HSA concentration was lower in the T1DM group (*p* = 0.0413). Statistically significant differences are indicated by * where *p* < 0.05, ** where *p* < 0.01 and *** where *p* < 0.001. Where no significant differences were observed (*p* > 0.05), it is denoted by ns.

In addition to collected demographic information, plasma concentration of triglycerides, cholesterol, LDL, HDL, HbA1c and HSA were measured and the cholesterol ratio was calculated subjects with T1DM and the controls. Summaries of this data are presented in Table 1, Fig. 1 and Table S1. The T1DM and control groups were matched for age and sex, but the T1DM group had a higher BMI (*p* = 0.0359). There was no difference in the concentrations of triglycerides, cholesterol, LDL, HDL and cholesterol ratio between the groups. In the T1DM group, HbA1c was higher (*p* < 0.0001) and HSA was lower (*p* = 0.0413) compared to controls.

**Table 1:** Demographic information on the T1DM and control groups. Where differences are significant, the *p*-value is provided. Where no significant difference was observed (p > 0.05), it is denoted by ns.

	Controls (*n* = 44)		T1DM subjects (n = 44)		Statistical significance
	Mean	SD	Mean	SD	*P* values
Age (years)	24.1	6.2	26.4	6.8	ns
Sex (% of male)	55	–	59	–	ns
Height (m)	1.73	0.10	1.73	0.10	ns
BMI (kg/m^2^)	23.1	3.0	24.6	3.5	0.0359
Numbers of smoking individuals	2	–	13	–	–
Numbers of macrovascular events	0	–	0	–	–
Numbers of microvascular events	0	–	1	–	–
Numbers of individuals with familial history of autoimmunity	11	–	16	–	–
Numbers of individuals with familial history of Huntington's disease	15	–	9	–	–
Diabetes duration (months)	–	–	125	103	–

### 3.2 T1DM-associated changes in FFA concentrations and plasma indices

**Fig. 2:**
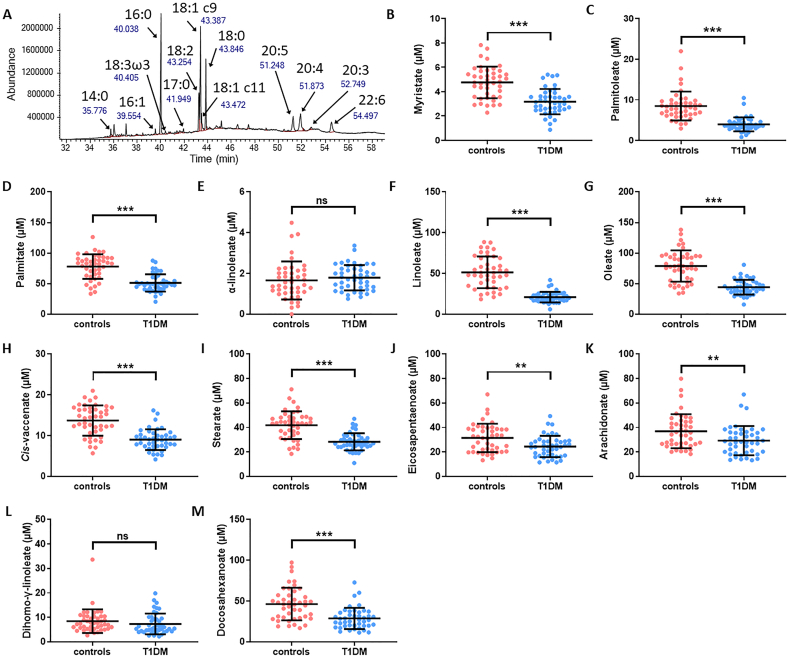
Plasma concentrations of major FFA species in T1DM and control individuals measured by GC–MS. A. Typical GC–MS chromatogram showing FAME separation. B-M. Plasma concentrations of B. myristate (14:0), C. palmitoleate (16:1), D. palmitate (16:0), E. α-linolenate (18:3), F. linoleate (18:2), G. oleate (18:1c9), H. *cis*-vaccenate (18:1c11), I. stearate (18:0), J. eicosapentaenoate (20:5), K. arachidonate (20:4), L. dihomo-γ-linolenate (20:3) and M. docosahexaenoate (22:6) are presented for subjects with T1DM and controls. Plasma FFA concentrations were reduced in the T1DM group compared to the control group (p < 0.0001 for myristate, linoleate, oleate, *cis*-vaccenate, stearate and docosahexaenoate, *p* = 0.0020 for eicosapentaenoate, *p* = 0.0068 for arachidonate), except for α-linolenate and dihomo-γ-linolenate, which were not significantly changed. Statistically significant differences are indicated by * where p < 0.05, ** where p < 0.01 and *** where p < 0.001. Where no significant differences were observed (p > 0.05), it is denoted by ns.

A FAME analysis was carried out on plasma samples from both T1DM and control groups to identify and quantify the major FFA species (Fig. 2 and Table S2). With the exception of α-linolenate (18:3) and dihomo-γ-linolenate (20:3), the concentrations of all major FFA species are lower in plasma taken from the T1DM group compared to controls: myristate (14:0; *p* < 0.0001), palmitoleate (16:1; p < 0.0001), palmitate (16:0; p < 0.0001), linoleate (18:2; p < 0.0001), oleate (18:1c9; *p* < 0.0001), *cis*-vaccenate (18:1c11; p < 0.0001), stearate (18:0; p < 0.0001), eicosapentaenoate (20:5; *p* = 0.0020), arachidonate (20:4; *p* = 0.0068) and docosahexaenoate (22:6, p < 0.0001).

**Fig. 3:**
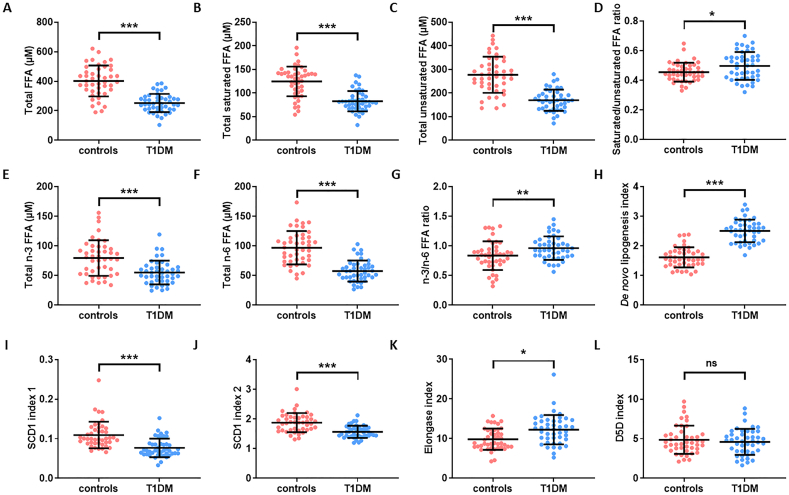
Plasma FFA indices in T1DM and control individuals. Plasma values are provided of A. Total plasma FFA concentration, B. total plasma concentration of saturated FFAs, C. total plasma concentration of unsaturated FFAs, D. saturated/unsaturated FFA ratio, E. total plasma concentration of n-3 FFAs, F. total plasma concentration of n-6 FFAs, G. n-3/n-6 FFA ratio, H. *de novo* lipogenesis index, I. SCD1 index 1, J. SCD1 index 2, K. elongase index, and L. D5D index are presented for T1DM and controls. Plasma concentration of total FFA, total saturated FFA, total unsaturated FFA, n-3 FFA and n-6 FFA and the SCD1 index 1 and SCD1 index 2 were reduced in the T1DM group (p < 0.0001 for all). The saturated/unsaturated FFA and n-3/n-6 ratios and the *de novo* lipogenesis and elongase indices were increased in the T1DM group (*p* = 0.0166, *p* = 0.0089, p < 0.0001 and *p* = 0.0008 respectively). The D5D index was unchanged. Statistically significant differences are indicated by * where p < 0.05, ** where p < 0.01 and *** where p < 0.001. Where no significant differences were observed (p > 0.05), it is denoted by ns.

Different plasma FFA indices were then calculated and compared between the T1DM and control groups (Fig. 3 and Table S3). Plasma concentration of total FFAs, total saturated FFAs, total unsaturated FFAs, total n-6 FFAs and total n-3 FFAs were lower in the T1DM group than in the controls (p < 0.0001 for all). The saturated/unsaturated FFA ratio and n-3/n-6 ratio were higher in the T1DM group (*p* = 0.0166 and *p* = 0.0089 respectively). The *de novo* lipogenesis index was calculated as the ratio of palmitate (16:0, the main product of lipogenesis) with linoleate (18:2, an essential FFA only found in the diet). The elongase index, which indicates elongase enzyme activity was calculated as the oleate/palmitoleate (18:1c9/16:1) ratio. The activity of stearoyl-CoA desaturase 1 (SCD1) was calculated using SCD1 indices 1 and 2, which reflect the palmitoleate/palmitate (16:1/16:0) ratio and the oleate/stearate (18:1c9/18:0) ratio, respectively. The delta-(5)-desaturase (D5D) index was calculated with the arachidonate/dihomo-γ-linolenate (20:4/20:3) ratio. The *de novo* lipogenesis index and the elongase index were higher (*p* < 0.0001 and *p* = 0.0008 respectively) and the SCD1 index 1 and 2 were lower (p < 0.0001 for both) in the T1DM group compared to the controls. The D5D index was unchanged between the groups.

### 3.3 Association between HbA1c concentration and plasma FFAs

**Fig. 4:**
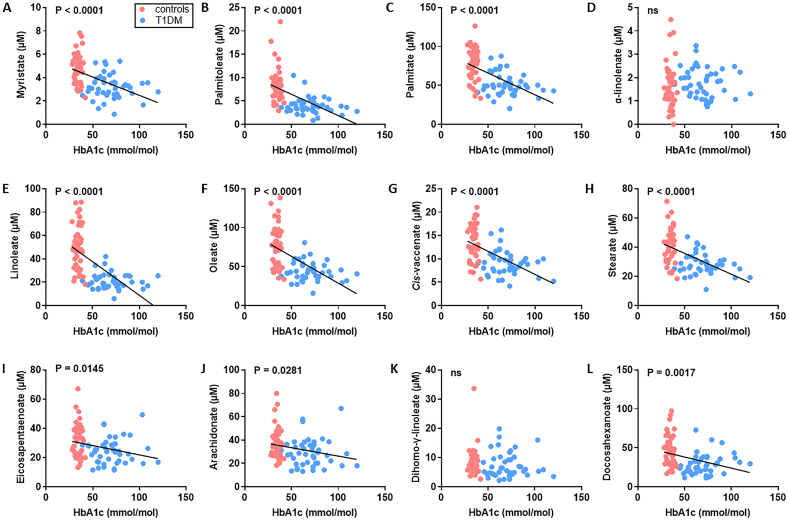
Associations between HbA1c concentration and plasma concentrations of major FFA species in subjects with T1DM and controls. A-L. Relationship between HbA1c and plasma concentrations of FFAs between subjects with T1DM and controls; A. myristate (14:0), B. palmitoleate (16:1), C. palmitate (C16:0), D. α-linolenate (18:3), E. linoleate (18:2), F. oleate (G18:1c9), G. *cis*-vaccenate (18:1c11), H. stearate (18:0), I. eicosapentaenoate (20:5), J. arachidonate (C20:4), K. dihomo-γ-linolenate (20:3) and L. docosahexaenoate (22:6). Blue circles represent samples from individuals from the T1DM group, red circles represent samples from individuals from the control group. With the exception of α-linolenate and dihomo-γ-linolenate, all plasma FFA concentrations negatively correlated with HbA1c (p < 0.0001 for myristate, palmitate, linoleate, oleate, *cis*-vaccenate and stearate, *p* = 0.0145 for eicosapentaenoate, *p* = 0.0281 for arachidonate and *p* = 0.0017 for docosahexanoate). Where correlations are significant, the *p*-value is provided. Where no significant correlation was observed (p > 0.05), it is denoted by ns.

**Fig. 5:**
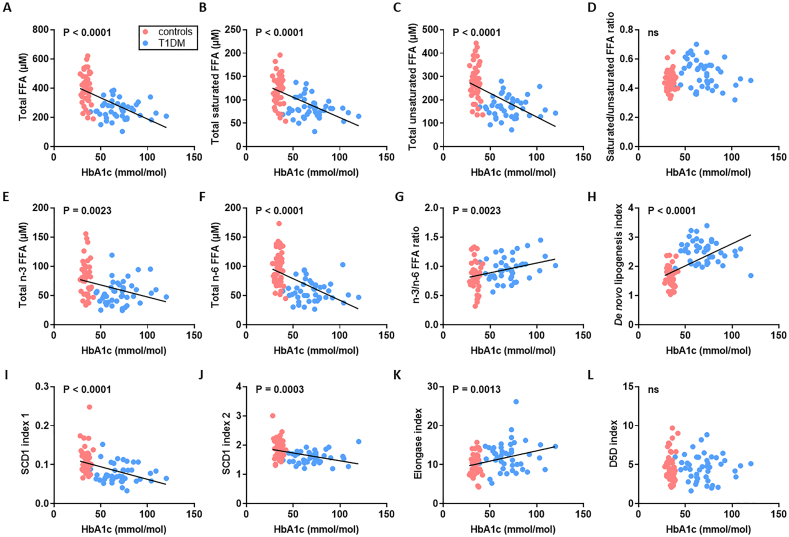
Associations between HbA1c concentration and different indices and FFA concentrations in T1DM and controls. Relationship between HbA1c concentration and A. total plasma FFA concentration, B. total plasma concentration of saturated FFAs, C. total plasma concentration of unsaturated FFAs, D. saturated/unsaturated FFA ratio, E. total plasma concentration of n-3 FFAs, F. total plasma concentration of n-6 FFAs, G. n-3/n-6 FFA ratio, H. *de novo* lipogenesis index, I. SCD1 index 1, J. SCD1 index 2, K. elongase index, and L. D5D index are presented for T1DM and controls. Blue circles represent samples from individuals from the T1DM group, red circles represent samples from individuals from the control group. In T1DM and controls, HbA1c concentration was negatively correlated with plasma concentrations of total FFAs, total saturated FFAs, total unsaturated FFAs, total n-3 FFAs and total n-6 FFAs and with SCD1 index1 and SCD1 index 2 (p < 0.0001, p < 0.0001, p < 0.0001, *p* = 0.0023, p < 0.0001, p < 0.0001 and *p* = 0.0003 respectively) and positively correlated with the n-3/n-6 FFA ratio, the *de novo* lipogenesis index and the elongase index (p = 0.0023, p < 0.0001 and *p* = 0.0013 respectively). Where correlations are significant, the p-value is provided. Where no significant correlation was observed (p > 0.05), it is denoted by ns.

We examined the relationship between HbA1c concentration and plasma concentrations of the major FFA species (Fig. 4 and Table S4 and S5). With the exception of α-linolenate (18:3) and dihomo-γ-linolenate (20:3), all plasma FFA concentrations negatively correlated with HbA1c (*p* < 0.0001 for myristate (14:0), palmitate (16:0), linoleate (18:2), oleate (18:1c9), *cis*-vaccenate (18:1c11) and stearate (18:0), *p* = 0.0145 for eicosapentaenoate (20:5), *p* = 0.0281 for arachidonate (20:4) and *p* = 0.0017 for docosahexanoate (22:6)). We then explored the relationships between HbA1c and the different FFA indices (Fig. 5). The plasma concentrations of total FFAs, saturated FFAs, unsaturated FFAs, n-6 FFAs and n-3 FFAs negatively correlated with HbA1c (*p* < 0.0001, p < 0.0001, p < 0.0001, *p* = 0.0023 and p < 0.0001 respectively). The saturated/unsaturated FFA ratio did not show any correlation with HbA1c but the n-3/n-6 ratio positively associated with HbA1c (p = 0.0023). The SCD1 index negatively associated with HbA1c whether measured using either palmitoleate/palmitate (16:1/16:0) or oleate/stearate ratios (18:1c9/18:0; p < 0.0001 and *p* = 0.0003 respectively). The D5D index was not associated with HbA1c but the elongase and *de novo* lipogenesis indices each positively correlated with HbA1c (*p* = 0.0013 and < 0.0001 respectively). We then assessed the relationship between HbA1c concentration and plasma concentrations of cholesterol, HDL, LDL and between HBA1c concentration and the cholesterol/HDL ratio and diabetes duration (Table S6). No associations between these respective parameters were found.

### 3.4 Assessment of age and sex-dependent associations with lipid concentrations and of diabetes duration with plasma FFA concentrations

The presence of sex or age-specific differences in the plasma concentrations of major FFA species was assessed. No differences in the plasma concentration of specific FFA species or in the indices monitored were found between males and females with T1DM (Fig. S1 and S2 and Table S7). There were no associations between age and the plasma concentration of major FFA species in T1DM, but the SCD1 index 1 positively correlated with age (*p* = 0.0098), while the elongase index negatively correlated with age (*p* = 0.0363; Fig. S3 and S4 and Table S8). In controls, plasma concentrations of palmitate (16:0), linoleate (18:2), oleate (18:1c9), *cis*-vaccenate (18:1c11) and stearate (18:0) were negatively correlated with age (*p* = 0.0006, *p* = 0.0037, *p* = 0.0014, *p* = 0.0003 and *p* = 0.0007 respectively; Fig. S5). In addition, total plasma FFA concentration, total plasma concentration of saturated, unsaturated and n-6 FFAs (but not n-3) were negatively correlated with age (*p* = 0.0008, *p* = 0.0005, *p* = 0.0018 and *p* = 0.0031 respectively), while the D5D index was positively correlated with age (*p* = 0.0057; Fig. S6). We examined the association between different lipids and the plasma concentrations of FFA species in T1DM (Table 2 and Fig. S7). LDL and the duration of diabetes were not correlated with any of the parameters. Cholesterol positively associated with palmitoleate (16:1) and linoleate (18:2) concentrations and the SCD1 index 1 (*p* = 0.0210, *p* = 0.0041 and *p* = 0.0479, respectively), and negatively associated with the *de novo* lipogenesis index (*p* = 0.0003). HDL positively associated with the plasma concentrations of myristate (14:0), palmitate (16:0), linoleate (18:2), *cis*-vaccenate (18:1c11), stearate (18:0), total FFAs and total saturated FFAs (*p* = 0.0372, *p* = 0.0321, *p* = 0.0007, *p* = 0.0090, *p* = 0.0124, *p* = 0.0455 and *p* = 0.0212 respectively) and negatively associated with the *de novo* lipogenesis index (*p* = 0.0258). The cholesterol ratio positively associated with the SCD1 index 1 (*p* = 0.0424).

**Table 2:** Associations between plasma lipid concentrations and plasma concentrations of major FFA species and FFA indices in T1DM. Where the correlations are significant (p < 0.05) the p values are provided; negative (-) and positive (+) correlations are indicated in parentheses. Where no significant correlation was observed (p > 0.05), it is denoted by ns.

	P values				
	Cholesterol	LDL	HDL	Cholesterol ratio	T1DM duration
Myristate; 14:0	ns	ns	0.0372(+)	ns	ns
Palmitoleate; 16:1	0.0210(+)	ns	ns	ns	ns
Palmitate; 16:0	ns	ns	0.0321(+)	ns	ns
α-linolenate; 18:3	ns	ns	ns	ns	ns
Linoleate; 18:2	0.0041(+)	ns	0.0007(+)	ns	ns
Oleate; 18:1c9	ns	ns	ns	ns	ns
*Cis*-vaccenate 18:1c11	ns	ns	0.0090(+)	ns	ns
Stearate; 18:0	ns	ns	0.0124(+)	ns	ns
Eicosapentaenoate; 20:5	ns	ns	ns	ns	ns
Arachidonate; 20:4	ns	ns	ns	ns	ns
Dihomo-γ-linolenate; 20:3	ns	ns	ns	ns	ns
Docosahexanoate; 22:6	ns	ns	ns	ns	ns
Total plasma FFA conc.	ns	ns	0.0455(+)	ns	ns
Total plasma conc. of saturated FFAs	ns	ns	0.0212(+)	ns	ns
Total plasma conc. of unsaturated FFAs	ns	ns	ns	ns	ns
Saturated FFAs/unsaturated FFAs	ns	ns	ns	ns	ns
Total plasma conc. of n-3 FFAs	ns	ns	ns	ns	ns
Total plasma conc. of n-6 FFAs	ns	ns	ns	ns	ns
n-3/n-6	ns	ns	ns	ns	ns
*De novo* lipogenesis index	0.0003(−)	ns	0.0258(−)	ns	ns
SCD1 index 1	0.0479(+)	ns	ns	0.0424(+)	ns
SCD1 index 2	ns	ns	ns	ns	ns
Elongase index	ns	ns	ns	ns	ns
D5D index	ns	ns	ns	ns	ns

## 4 Discussion

The concentrations of specific plasma FFA species in individuals with T1DM has not been fully investigated before. Indeed, only one report of alterations in plasma FFA levels in T1DM has been made: a metabolomic study by Dutta et al. comparing individuals with T1DM with “good” or “bad” glycaemic controls (14 and 15 individuals, respectively) and controls (14 matched with the individuals with T1DM and good glycaemic control and 15 matched with individuals with T1DM and bad glycaemic control) [19]. Using untargeted metabolomic analysis, this study found that individuals with T1DM with bad glycaemic control have reduced FFA levels. The authors then used targeted analysis on myristate (14:0), palmitoleate (16:1), palmitate (16:0), α-linolenate (18:3), linoleate (18:2), elaidate (18:1 *trans*), oleate (18:1 *cis*), stearate (18:0) and arachidonate (20:4) and they found elevated levels of oleate, elaidate and stearate in individuals with T1DM and good glycaemic control compared to healthy controls, while unchanged plasma FFA levels were found in individuals with T1DM and bad glycaemic control compared to healthy control. Here, FFAs were extracted from plasma taken from 45 individuals with T1DM and 45 controls and their concentrations were determined using GC–MS. Our cohort was age and sex-matched, but BMI was slightly higher in the T1DM group. HSA concentration was also lower in the T1DM group compared to the controls, which is in agreement with a study that found that insulin withdrawal in women with T1DM leads to reduced HSA synthesis [20]. Unchanged lipid (HDL, LDL, cholesterol and triglycerides) levels in individuals with T1DM have been reported before [21], and are thought to be the result of good glycaemic control [22]. The concentrations of total FFAs, and of most FFA species measured, were found to be reduced in the T1DM group compared to controls, in accord with the previous metabolomics study by Dutta et al. [19].

In order to fully understand our results, different fatty acid (FA) indices were calculated to indicate the activities of various enzyme classes involved in FA metabolism. The increase in n-3/n-6 ratio in the T1DM individuals was unexpected, as n-3 FAs in the sn2 position form a reservoir for the production of anti-inflammatory mediators [23]. In addition, a deficiency in n-3 FFAs may heighten inflammatory reactions and increase the risk of developing auto-immune diseases such as T1DM [24]. On the contrary, n-6 FAs form pro-inflammatory molecules, and so n-6 FFAs may have been expected to be elevated in T1DM. However, this view may be too simplistic as n-6 FFAs are also precursors of anti-inflammatory molecules and there is no epidemiological evidence suggesting that an increase intake of the n-6 FAs linoleate (18:2) and arachidonate (20:4) promotes inflammation. In addition, despite previous reports on the benefits of n-3 supplementation in children at risk of developing T1DM [[24], [25], [26], [27]], a recent meta-analysis on the effect of n-3 and n-6 FA supplementation in pregnancy or early life of children found no positive effect on T1DM occurrence [28].

Total saturated and unsaturated FFAs and the saturated/unsaturated FFA ratio were then calculated. The fact that the saturated/unsaturated ratio increased was expected, as this ratio and the nature of the unsaturated FAs in cellular membranes are important for the structure, fluidity and functioning of the cells. A study performed in children with one parent with T1DM has shown that those who then subsequently develop T1DM themselves had a higher proportion of polyunsaturated FAs in their phospholipids possibly due to altered lipid absorption [3]. The fact that the proportion of saturated FFAs is higher in plasma in our cohort could be due to differential absorption of FFAs by the cells (and incorporation into phospholipids) or to a difference in lipid profiles at different ages (our cohort being older).

The *de novo* lipogenesis index is calculated as the ratio of palmitate (16:0, the main FFA synthesised by *de novo* lipogenesis) and linoleate (18:2, an essential FFA only obtained from the diet). The increase in this index in T1DM individuals compared to controls could be explained by an increase in lipogenesis, though it is unlikely as palmitate itself has a reduced concentration in T1DM. In addition, a study conducted on 9 individuals with T1DM and 9 controls found unchanged levels of fasting or postprandial hepatic *de novo* lipogenesis [21]. Thus, the increase in lipogenesis index we measured was more probably due to either a reduced FFA intake from the diet or the reduced absorption of dietary FFAs. Changes in the dietary habits of individuals with T1DM after diagnosis is likely, as control of the diet is an important axis of T1DM management (NICE guidelines from the UK [NG17] and [NG18] [29,30]). Nevertheless, the results of studies investigating the dietary habits of individuals with T1DM compared to controls vary geographically. Indeed, while individuals in Spain have been noted to have on average better adherence to the Mediterranean Diet (low intake of animal fat, high intake of fruits, vegetables, grains, olive oil and moderate intake of fish, poultry and red wine) compared to controls [31], a T1DM cohort from the USA consumed more fat and saturated fat compared to controls [32], while a T1DM cohort from China had a higher energy percentage from fat than controls [33]. Diet was not monitored in our UK cohort so it is not possible to evaluate the degree to which dietary differences may have influenced our results. However, altered absorption of dietary FFAs and altered lipid metabolism are likely to play a major role as FFAs have been noted to be the main energy source of the heart in T1DM individuals, replacing glucose [34].

In the T1DM group, SCD1 indices 1 and 2 decreased, meaning that a smaller proportion of palmitate (16:0) and stearate (18:0) were desaturated in T1DM individuals (explaining the higher proportion of saturated FFAs), while the elongase index increased compared to controls, which meant that more palmitoleate (16:1) was converted into oleate (18:1c9). The D5D index was unchanged between the groups, implying that the rate of conversion dihomo-γ-linolenate (20:3) into arachidonate (20:4) was unchanged. Another study also showed reduced desaturation in T1DM, but contrary to our study also found elongation to be reduced [35]. This reduction of elongation may be explained by a difference in methodology leading to different enzymes being studied (reduced linoleate/arachidonate (18:2/20:4) ratio in serum cholesterol, serum phospholipids and erythrocytes or unchanged in serum triglycerides and in platelets, *versus* our increased oleate/palmitoleate (18:1c9/16:1) ratio in plasma FFAs).

The influence of sex, age, glucose control, diabetes duration, cholesterol, LDL and HDL concentration on plasma FFA concentrations and lipid indices were examined. No sex difference in plasma FFA concentrations or lipid indices were observed in the T1DM group. This is in contrast to previously reported differences in lipid metabolism between men and women [36], but in accord with a previous study in healthy individuals that found no difference in most plasma FFA species concentrations between sexes and only a higher concentrations of docosahexanoate (22:6) and total n-3 FFAs in women compared to men [37]. In healthy individuals, age was negatively correlated with the plasma concentrations of most FFAs and was positively correlated with the D5D index. In individuals with T1DM these associations were not present; the only effects with age were a positive correlation with SCD1 index 1 but not SCD1 index 2, and a negative correlation with the elongase index. Day-long postprandial FFA concentrations have been reported to be lower in older non-diabetic individuals, which is in accord with our results in the control group [38]. The HbA1c concentration reflects the degree of control of plasma glucose concentration, a direct or indirect controller of a wide range of metabolic processes, including lipid metabolism [3,4]. The metabolomic study by Dutta et al. confirmed the importance of glycaemic control in lipid metabolism by revealing metabolic changes in individuals with T1DM who had poor glycaemic control, compared to those with good glycaemic control [19]. Thus, it is unsurprising to find that HbA1c concentration associates with the plasma concentration of most of the major FFA species. However, just as a diet high in fat is associated with poor glycaemic control [39], it would be expected that HbA1c levels would be positively associated with plasma FFA levels in T1DM. Here HbA1c was negatively correlated with the concentrations of the different plasma FFAs. In addition, the other changes observed in the T1DM group compared to the control group were also increased with poor glycaemic control, with the exception of the saturated/unsaturated FFA ratio which was not associated with HbA1c level. Thus, either the expression or the function of the elongase and desaturase enzymes is likely to be regulated by plasma glucose levels. However, T1DM itself also affects their expression, with SCD1 having a reduced expression in the kidney in NOD mice with autoimmune diabetes [40]. It is unknown if some individuals with T1DM possess genetic mutations that result in altered desaturase or elongase expression or activity.

Associations have previously been found between some specific FAs found in phospholipids and the concentrations of cholesterol, HDL or LDL: cholesterol concentration positively correlated with eicosapentaenoate concentration (20:5) and HDL concentration with α-linolenate (18:3) and eicosapentaenoate concentrations; LDL concentration positively correlated with eicosapentaenoate and docosahexaenoate (22:6) concentrations and negatively correlated with linoleate (18:2) concentrations, while no association was found with the concentrations of dihomo-γ-linolenate (20:3) and arachidonate (20:4) [41]. In our study, only minimal associations were found: the HDL concentration was positively correlated with the concentrations of total plasma FFAs, total saturated FFAs, and some FFAs (myristate (14:0), palmitate (16:0), linoleate (18:2), *cis*-vaccenate (18:1c11), stearate (18:0)), while the cholesterol concentration was positively correlated with the SCD1 index 1 and with palmitoleate (16:1) and linoleate (18:2) FFA concentrations and negatively correlated with the *de novo* lipogenesis index. The cholesterol ratio was positively correlated with the SCD1 index 1, while neither the diabetes duration nor the LDL concentration appeared to have any influence on plasma FFA concentrations. Thus, plasma HDL concentration and to a lesser degree, plasma cholesterol concentration seem to be closely associated with plasma FFA concentrations. While HDL concentration did not differ between our T1DM and control groups, HDL function is known to be reduced in T1DM [42].

In conclusion, this study greatly enhances previous knowledge of how plasma FFA metabolism is altered in T1DM. These alterations include reductions in the plasma concentrations of most major FFA species, increases in saturated/unsaturated FFA ratios, n-3/n-6 FFA ratio, *de novo* lipogenesis index and elongase index and decreases in SCD1 indices 1 and 2, with those differences most prevalent in those with poor glycaemic control. Current studies prevent us from asserting the extent to which diet is responsible for the altered lipid profile we observe or if there is an altered absorption of dietary FFAs in T1DM. *De novo* lipogenesis itself is unlikely to be increased, but lipogenesis products are more predominant within the lipid profile in T1DM, possibly due to altered diet or alerted dietary absorption of FFAs. Thus, while part of these results could potentially reflect the dietary changes, they also reflect strong alterations in lipid metabolism, particularly in FA desaturation and elongation. The alterations in lipid metabolism may have important consequences for metabolic regulation, in particular for inflammation, cellular function and oxidative stress management, through alteration of cellular membrane composition, activation of FA receptors and precursor availability for the synthesis of signalling molecules. The results highlighted by our study are important, as differences in lipid metabolism may be a key driver of complications in individuals with the disease. Therefore, these data may be useful not only in increasing our understanding of T1DM but also for management of the disease.

## Funding

This research was funded by the 10.13039/501100000274British Heart Foundation [grant numbers PG/15/9/31270, FS/15/42/3155].

## CRediT authorship contribution statement

**Amélie I.S. Sobczak:** Conceptualisation, Methodology, Formal analysis, Investigation, Data curation, Writing - original draft, Writing - review & editing, Visualisation. **Samantha J. Pitt:** Writing - review & editing, Funding acquisition. **Terry K. Smith:** Conceptualisation, Methodology, Validation, Resources, Writing - review & editing, Supervision. **Ramzi A. Ajjan:** Conceptualisation, Resources, Writing - review & editing, Supervision, Project administration, Funding acquisition. **Alan J. Stewart:** Conceptualisation, Methodology, Resources, Writing - original draft, Writing - review & editing, Supervision, Project administration, Funding acquisition.

## Declaration of competing interest

The authors declare that they have no known competing financial interests or personal relationships that could have appeared to influence the work reported in this paper.
